# Blood Profile of Cytokines, Chemokines, Growth Factors, and Redox Biomarkers in Response to Different Protocols of Treadmill Running in Rats

**DOI:** 10.3390/ijms21218071

**Published:** 2020-10-29

**Authors:** Elżbieta Supruniuk, Mateusz Maciejczyk, Anna Zalewska, Jan Górski, Adrian Chabowski

**Affiliations:** 1 Department of Physiology, Medical University of Bialystok, 2C Mickiewicza Street, 15-222 Bialystok, Poland; adrian@umb.edu.pl; 2 Department of Hygiene, Epidemiology and Ergonomics, Medical University of Bialystok, 2C Mickiewicza Street, 15-233 Bialystok, Poland; mat.maciejczyk@gmail.com; 3 Experimental Dentistry Laboratory, Medical University of Bialystok, 24a M. Sklodowskiej-Curie Street, 15-274 Bialystok, Poland; azalewska426@gmail.com; 4 Department of Medical Sciences, Lomza State University of Applied Sciences, 18-400 Lomza, Poland; gorski@umb.edu.pl

**Keywords:** exercise, inflammation, cytokines, chemokines, oxidative stress

## Abstract

Both positive and negative aspects of sport performance are currently considered. The aim of our study was to determine time- and intensity-dependent effects of a single exercise bout on redox and inflammatory status. The experiment was performed on 40 male Wistar rats subjected to treadmill running for 30 min with the speed of 18 m/min (M30) or 28 m/min (F30), or for 2 h with the speed of 18 m/min (M120). Immunoenzymatic and spectrophotometric methods were applied to assess the levels of pro-inflammatory and anti-inflammatory cytokines, chemokines, growth factors, the antioxidant barrier, redox status, oxidative damage products, nitrosative stress, and their relationships with plasma non-esterified fatty acids. Treadmill running caused a reduction in the content of monocyte chemoattractant protein-1 (MCP1) and nitric oxide (M30, M120, F30 groups) as well as macrophage inflammatory protein-1α (MIP-1α) and regulated on activation, normal T-cell expressed and secreted (RANTES) (M30, F30 groups). We also demonstrated an increase in catalase activity as well as higher levels of reduced glutathione, advanced oxidation protein products, lipid hydroperoxides, malondialdehyde (M30, M120, F30 groups), and advanced glycation end products (F30 group). The presented findings showed the activation of antioxidative defense in response to increased reactive oxygen species’ production after a single bout of exercise, but it did not prevent oxidative damage of macromolecules.

## 1. Introduction

Regular physical activity has been acknowledged a pivotal component of a healthy lifestyle and recommended in the management and treatment of atherosclerosis, insulin resistance, type 2 diabetes, cardiovascular diseases, cognitive disorders, and cancer [1]. Collectively, the multi-systemic benefits of aerobic exercise enable to extend life expectancy by 3–10%, delay the start of morbidity, and diminish all-cause mortality risk by 30–40% [2]. Acute training triggers alterations in the cellular status of Ca^2+^ and superoxide anion (O_2_^•^^−^), the ratio of oxidized and reduced forms of nicotinamide adenine dinucleotide (NAD^+^/NADH), AMP/ATP ratio, as well as influences the release of numerous hormones and neurotransmitters [3]. Exercise is an initiator of signal-transmitting cascades involved in the regulation of glucose tolerance and cellular repair as well as the transcriptional modulation of redox and cytokine signaling [4]. For instance, exercise-induced nuclear factor-like 2 (Nrf2) regulates nuclear factor κB (NF-κB)-dependent mechanisms that control downstream antioxidant and inflammatory responses [5,6]. The effects of training also rely on reduced expression of Toll-like receptors (TLRs) on monocytes and macrophages that subsequently diminish pro-inflammatory cytokines’ production [1]. 

Contrary to regular physical activity, an intense and exhaustive exercise could trigger negative consequences in terms of health that result in oxidative damages to skeletal muscles at cellular and tissue levels. Especially, high energy demands and oxygen consumption are connected with increased number of electrons supplied to the respiratory chain and reactive oxygen (ROS) and nitrogen (RNS) species’ generation [7]. Muscle injury is also linked with specific leukocyte subsets’ infiltration [8] and interactions between released metmyoglobin/methemoglobin with peroxides [9], culminating in the release of inflammatory mediators and ROS/RNS. Free radicals could also serve as signaling molecules accelerating inflammation [10,11]. Moreover, hypoperfusion of other internal organs and heat induction could aggravate the degree of stress response [12]. Prolonged exposure to cytokines and free radicals becomes destructive and underlies the pathophysiology of chronic disorders, such as diabetes, cancer, or cardiovascular diseases [13,14,15]. 

There are only a few studies debating on a role of a single session of exercise in the interactions among oxidative stress and inflammation. For instance, a single-bout endurance training reduced IL-6/IL-10 ratio and monocyte chemoattractant protein 1 (MCP1) content in a healthy man [16] or increased IL-17 level in trained rats [17]. Another study found that six 10-s sprints did not affect plasma protein carbonyls (PC) and malondialdehyde (MDA) in anaerobically trained men [18]. In the following research we applied three standardized aerobic exercise protocols to obtain a comprehensive insight into the association between exercise intensity and duration and systemic inflammatory and redox responses. Particularly, we investigated the effects of physical activity on the level of pro- and anti-inflammatory cytokines, chemokines, and growth factors as well as redox biomarkers (i.e., enzymatic and non-enzymatic antioxidant barrier, redox status, oxidative damage to proteins and lipids, and nitrosative stress).

## 2. Results

### 2.1. Serum Concentrations of Cytokines in Response to Treadmill Running

**Figure 1:**
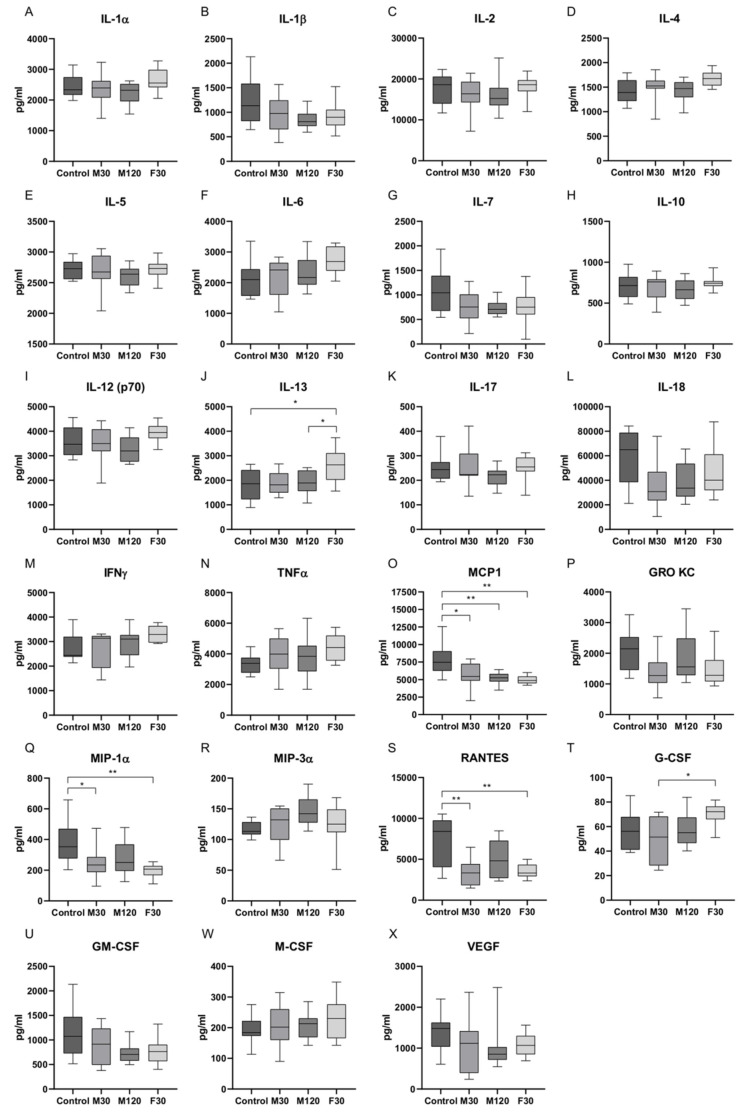
Serum expression of cytokines in control rats and in response to different protocols of treadmill running: (**A**) interleukin-1α, (**B**) IL-1β, (**C**) IL-2, (**D**) IL-4, (**E**) IL-5, (**F**) IL-6, (**G**) IL-7, (**H**) IL-10, (**I**) IL-12 (p70), (**J**) IL-13, (**K**) IL-17, (**L**) IL-18, (**M**) interferon γ, (**N**) tumor necrosis factor α, (**O**) monocyte chemoattractant protein-1, (**P**) growth-regulated oncogene/keratinocyte chemoattractant, (**Q**) macrophage inflammatory protein-1α, (**R**) macrophage inflammatory protein-3α, (**S**) regulated on activation, normal T-cell expressed and secreted, (**T**) granulocyte colony-stimulating factor, (**U**) granulocyte-macrophage colony stimulating factor, (**W**) macrophage colony-stimulating factor, and (**X**) vascular endothelial growth factor. The values (pg mL^−1^) are expressed as median (minimum-maximum) based on 10 independent determinations. ANOVA followed by a post hoc Tukey’s test or Kruskal–Wallis and a post hoc Dunn’s tests were applied to determine significant differences. * *p* < 0.05, ** *p* < 0.01; M30, rats running on a treadmill at the speed of 18 m/min for 30 min; M120, rats running on a treadmill at the speed of 18 m/min for 120 min; F30, rats running on a treadmill at the speed of 28 m/min for 30 min.

Cytokines are a diverse family of small, secreted proteins with pleiotropic functions [19], while their kinetics are early indicators of perturbation to the immune system [20]. In order to better define the immune response to a single exercise bout, rats were subjected to treadmill running for 30 min with the speed of 18 m/min (M30) or 28 m/min (F30) or for 2 h with the speed of 18 m/min (M120). There were no statistically significant differences in the level of pro-inflammatory cytokines, such as IL-1α, IL-6, IL-12 (p70), IL-17, interferon γ (IFNγ), and tumor necrosis factor α (TNFα) after all the implemented exercise protocols as compared to the control group (Figure 1). Nevertheless, a tendency toward diminished IL-1β and IL-7 was noticed in M120 group (−31%, −31%, M120 vs. control, respectively, *p* > 0.05; Figure 1B,G).

In the case of anti-inflammatory cytokines, there was a substantial increase in the content of IL-13 in F30 group (+44%, +38%, F30 vs. control and M120, respectively, *p* < 0.05; Figure 1J).

Among chemokines, a significant drop in MCP1 amount was observed in all the studied groups as compared to control (−29%, −34%, −37%, M30, M120, and F30 vs. control, respectively, *p* < 0.05; Figure 1O). A reduced contents of macrophage inflammatory protein-1α (MIP-1α) and regulated on activation, normal T-cell expressed and secreted (RANTES) were demonstrated for M30 and F30 groups (MIP-1α: −36%, −49%, RANTES: −53%, −52%, M30 and F30 vs. control, respectively, *p* < 0.05). In the case of M120 group, only trends towards diminished MIP-1α and RANTES levels were observed (−28%, −31%, M120 vs. control, respectively, *p* > 0.05; Figure 1Q,S). A slightly decreased amount of growth-regulated oncogene/keratinocyte chemoattractant (GRO KC) was also demonstrated (−34%, −29%, M30 and F30 vs. control, respectively, *p* > 0.05; Figure 1P).

There was a significant difference in granulocyte colony-stimulating factor (G-CSF) level between intense and moderate run for 30 min (+42%, F30 vs. M30, *p* < 0.05; Figure 1T). Additionally, a tendency toward reduced granulocyte-macrophage colony stimulating factor (GM-CSF) (−23%, −35%, −32%, M30, M120 and F30 vs. control, respectively, *p* > 0.05) content in response to exercise protocols was revealed (Figure 1U).

### 2.2. Enzymatic and Non-Enzymatic Antioxidants, Total Antioxidant/Oxidant Status, Oxidative Damage Products, and Nitrosative Stress in Plasma or Serum of Rats Subjected to Treadmill Running

**Figure 2:**
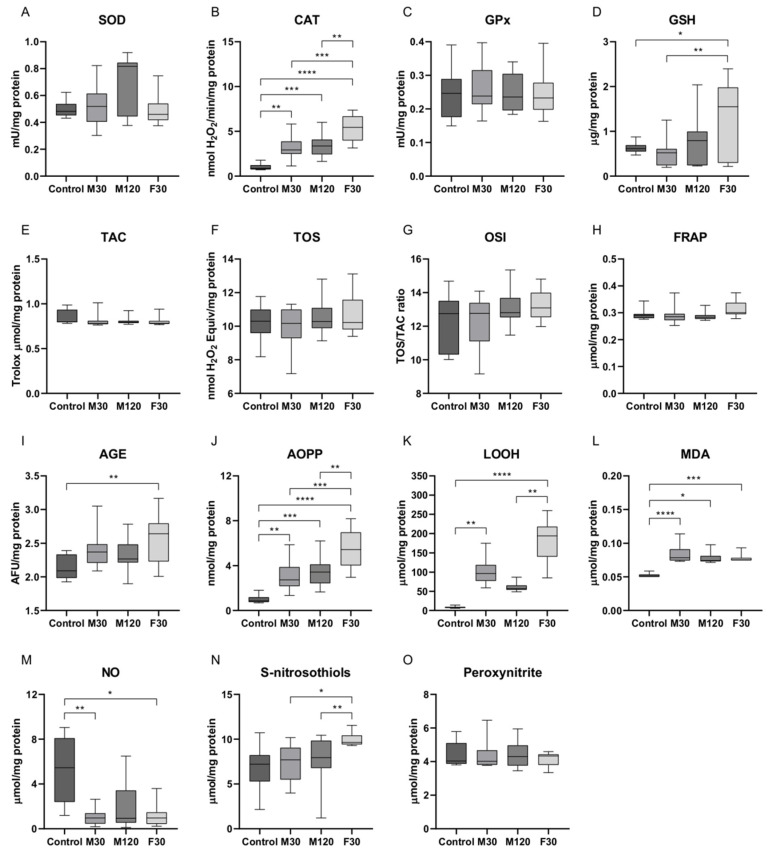
Redox biomarkers in response to different protocols of treadmill running: (**A**) superoxide dismutase, (**B**) catalase, (**C**) glutathione peroxidase, (**D**) reduced glutathione, (**E**) total antioxidant capacity, (**F**) total oxidant status, (**G**) oxidative stress index, (**H**) ferric reducing ability of plasma, (**I**) advanced glycation end products, (**J**) higher advanced oxidation protein products, (**K**) lipid hydroperoxides, (**L**) malondialdehyde, (**M**) nitric oxide, (**N**) S-nitrosothiols, and (**O**) peroxinitrite. The values are expressed as median (minimum-maximum) based on 10 independent determinations. ANOVA followed by a post hoc Tukey’s test or Kruskal–Wallis and a post hoc Dunn’s tests were applied to determine significant differences. * *p* < 0.05, ** *p* < 0.01, *** *p* < 0.001, **** *p* < 0.0001; M30, rats running on a treadmill at the speed of 18 m/min for 30 min; M120, rats running on a treadmill at the speed of 18 m/min for 120 min; F30, rats running on a treadmill at the speed of 28 m/min for 30 min.

Similarly to cytokines, the pattern of exercise-mediated changes in redox markers is highly variable and reflects the degree of cellular stress accompanying different intensities and durations of exercise [21]. In our study, the activity of catalase (CAT) markedly increased in groups that underwent all exercise programs as compared to control (+2-fold, +2.4-fold, +4.3-fold, M30, M120, and F30 vs. control, respectively, *p* < 0.05). Moreover, CAT activity was significantly higher in F30 group as compared to rats subjected to moderate-intensity running (+73%, +55%, F30 vs. M30 and M120, respectively, *p* < 0.05; Figure 2B). The activity of other enzymatic antioxidants, i.e., superoxide dismutase (SOD) and glutathione peroxidase (GPx), did not change significantly after treadmill running (Figure 2A,C). In F30 group, an increase in the plasma content of reduced glutathione (GSH) was demonstrated (+109%, +161%, +106%, F30 vs. control, M30, and M120, respectively, *p* < 0.05, Figure 2D).

The parameters of total antioxidant/oxidant status, i.e., total antioxidant capacity (TAC), total oxidant status (TOS), oxidative stress index (OSI), and ferric reducing ability of plasma (FRAP), remained relatively constant between the studied groups (Figure 2E–H). 

Macromolecules’ and cell structures’ modifications are one of the major outcomes of ROS/RNS-mediated injury [13]. We showed that the concentration of advanced glycation end products (AGE) was elevated in F30 group as compared to the control animals (+22%, F30 vs. control, *p* < 0.05; Figure 2I). Rats from all the running groups had substantially higher advanced oxidation protein products (AOPP) amount than sedentary control (+2.2-fold, +3-fold, +5.3-fold, M30, M120, and F30 vs. control, respectively, *p* < 0.05). Additionally, increasing the speed caused more pronounced changes in AOPP content as compared to other exercise protocols (+99%, +58%, F30 vs. M30 and M120, respectively, *p* < 0.05, Figure 2J). Rats that underwent training were also characterized by an increased concentration of lipid hydroperoxides (LOOH) as compared to the control (+11-fold, +6-fold, +24-fold, M30, M120, and F30 vs. control, respectively, *p* < 0.05). These changes were the highest in F30 group (+3-fold, F30 vs. M120, *p* < 0.05; Figure 2K). A single bout of training coincided with a significant rise in MDA level in animals’ plasma as compared to control (+54%, +46%, +48%, M30, M120, and F30 vs. control, respectively, *p* < 0.05; Figure 2L). 

Furthermore, treadmill running evoked a marked reduction in nitric oxide (NO) level, but without significant differences between the speed and time of activity (−82%, −82%, M30 and F30 vs. control, respectively, *p* < 0.05; −83%, M120 vs. control, *p* > 0.05; Figure 2M). An exercise intensity-dependent effect was observed for S-nitrosothiols level since it was markedly elevated in F30 group as compared to other running protocols (+25%, +21%, F30 vs. M30 and M120, respectively, *p* < 0.05; Figure 2N), while peroxynitrite content did not change significantly (Figure 2O).

### 2.3. Plasma Content and Composition of Free Fatty Acids (FFA) in Rats Subjected to Treadmill Running

Exercise has been shown to modulate the activity of various enzymes responsible for lipolysis (i.e., ↑ adipose triglyceride lipase and hormone sensitive lipase, ↓ lipoprotein lipase) [22]. In line with this, elevated content of plasma FFA was noticed in all training groups (+60%, +1.9-fold, +1.2-fold, M30, M120, and F30 vs. control, respectively, *p* < 0.05). Moreover, a longer duration of exercise led to substantially higher increase in FFA level as compared to 30 min of running (+79%, M120 vs. M30, *p* < 0.05, Table 1) [23].

### 2.4. Correlations

**Figure 3:**
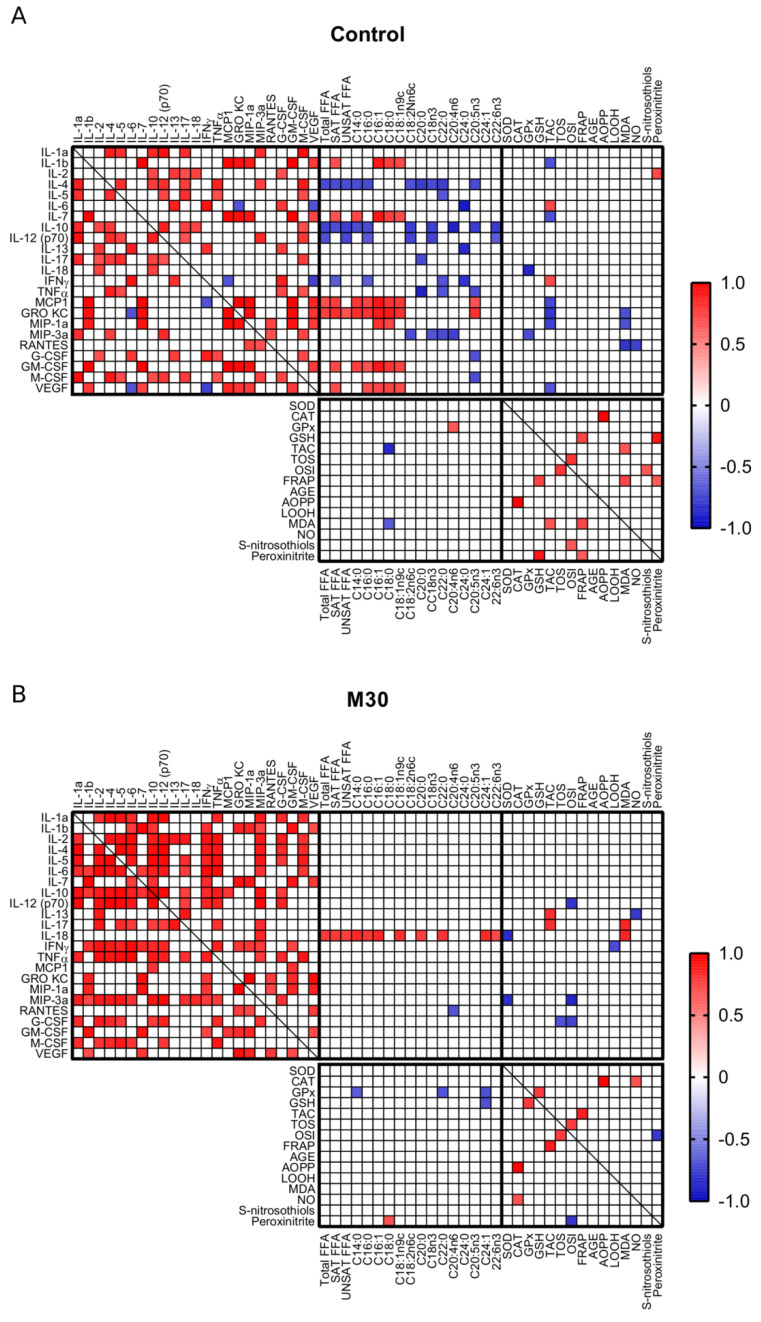

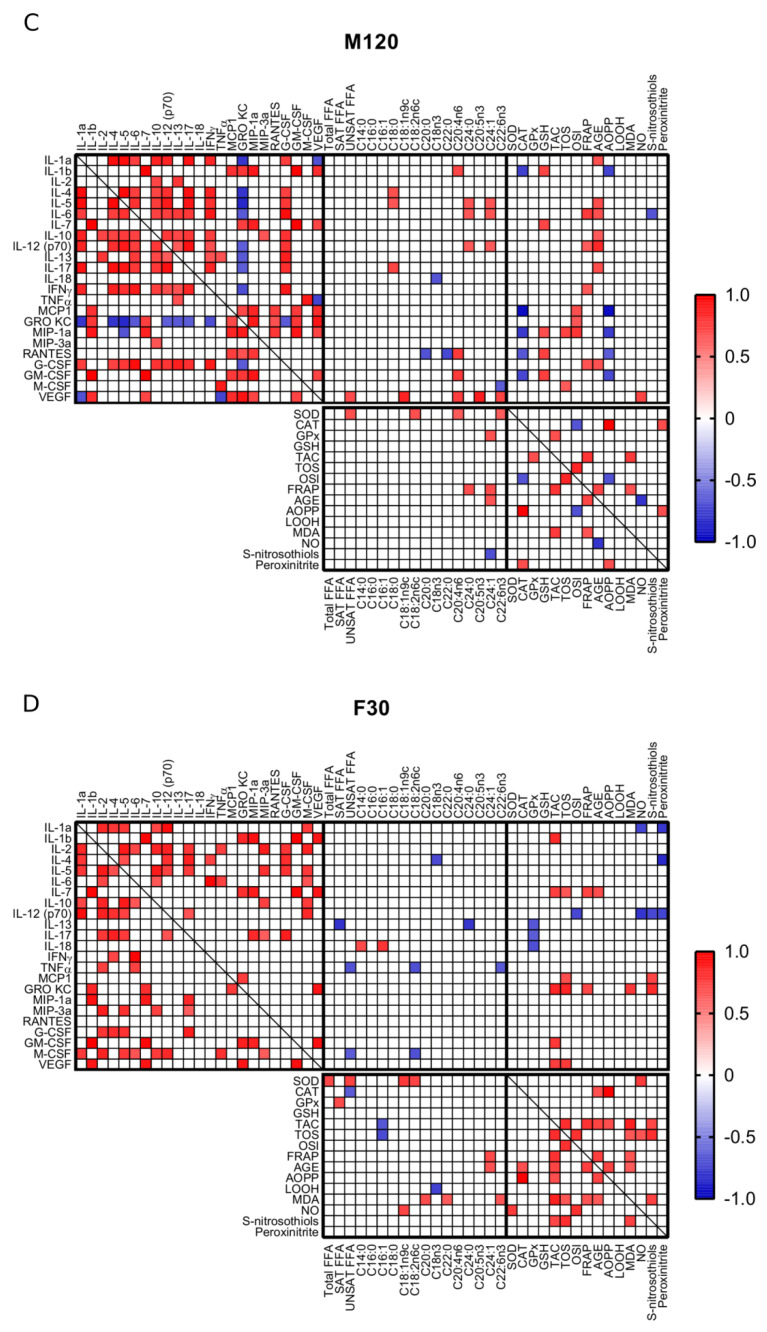
Correlations between serum cytokines, plasma free fatty acids, and oxidative stress markers in control rats (**A**) and in response to different protocols of treadmill running: (**B**) M30, (**C**) M120, (**D**) F30. The depicted values (*p* < 0.05) are based on the Pearson correlation coefficient. M30, rats running on a treadmill at the speed of 18 m/min for 30 min; M120, rats running on a treadmill at the speed of 18 m/min for 120 min; F30, rats running on a treadmill at the speed of 28 m/min for 30 min.

In all the studied groups, we observed positive correlations between pro- (i.e., IL-1α) and anti-inflammatory cytokines (i.e., IL-4, IL-5, IL-10, IL-12 (p70)). Additionally, serum level of IL-12 (p70) positively correlated with IL-4 and IL-5, while IL-17 with IL-5. There were also positive correlations between chemokines (i.e., GRO KC and MIP-1α) and growth factors (i.e., GM-CSF and vascular endothelial growth factor (VEGF)) with IL-1β and IL-7 in all the studied conditions. Prolonged (2 h) running was connected with negative correlation of GRO KC with several cytokines (i.e., IL-1α, IL-4, IL-5, IL-12 (p70), IL-13, IL-17, and IFNγ), unobserved in the other groups (Figure 3A–D). 

Several studies indicate that FFA influence cytokines and redox balance, pointing to a reduced content of IL-10, IL-12(p70), IFN-γ, and MCP1, and elevated amount of IL-2 and IL-18 in whole blood human samples exposed to FFA [24] as well as higher levels of redox biomarkers (i.e., catalase, MDA) caused by a high-fat diet in rats [25]. We examined whether such a relationship was noticed due to a single training session in rodents. In the control group, there were significant positive correlations between total FFA level with MCP1 and GRO KC expression (*r* = 0.69, *r* = 0.739, respectively, *p* < 0.05). In the case of saturated free fatty acids, correlations were noticed with IL-1β, IL-7, MCP1, GRO KC, GM-CSF, and VEGF (*r* = 0.68, *r* = 0.673, *r* = 0.761, *r* = 0.825, *r* = 0.706, *r* = 0.696, respectively, *p* < 0.05; Figure 3A). Negative relationships were demonstrated between IL-4 as well as IL-10 and total, saturated, and unsaturated fatty acid amount (IL-4: *r* = −0.712, *r* = −0.699, *r* = −0.708; IL-10: *r* = −0.738, *r* = −0.698, *r* = −0.751, respectively, *p* < 0.05), while for IL-12 (p70) the correlations were revealed with total and unsaturated fatty acids (*r* = −0.688, *r* = −0.722, respectively, *p* < 0.05; Figure 3A).

In M30 group, there were noticeable positive relationships between IL-18 and total, saturated, and unsaturated fatty acids (*r* = 0.771, *r* = 0.774, *r* = 0.756, respectively, *p* < 0.05; Figure 3B). Unsaturated species level was positively correlated with VEGF expression in M120 group (*r* = 0.702, *p* < 0.05; Figure 3C) and negatively correlated with TNFα and macrophage colony-stimulating factor (M-CSF) in F30 group (*r* = −0.676, *r* = −0.641; Figure 3D).

With the increased duration and speed of running, correlations between SOD and total (F30: *r* = 0.718, *p* < 0.05) or unsaturated (M120: *r* = 0.689, F30: *r* = 0.736, *p* < 0.05) non-esterified fatty acids were noticed (Figure 3C,D). 

Similarly, in exercise groups there was an increasing number of positive correlations between the redox biomarkers. Especially, in both M120 and F30 groups, TAC and FRAP (M120: *r* = 0.791, F30: *r* = 0.8, *p* < 0.05) and AGE and FRAP (M120: *r* = 0.723, F30: *r* = 0.748, *p* < 0.05) as well as TAC and MDA (M120: *r* = 0.765, F30: *r* = 0.869, *p* < 0.05) correlated with each other (Figure 3C,D). There were no permanent relationships between cytokines and oxidative parameters across the studied groups (Figure 3A–D).

## 3. Discussion

Given the population aging and constantly increasing global burden of chronic disorders, new strategies to decelerate organ deterioration and to provide systemic homeostatic control are sought. Herein, we aimed to compare blood cytokine and oxidative responses to three different protocols of treadmill running in rats with respect to the time of intervention (i.e., 30 min or 2 h) and its intensity (i.e., speed of run set on 18 m/min or 28 m/min).

We demonstrated that serum levels of pro- and anti-inflammatory cytokines were rather constant between sedentary and subjected-to-a-single-bout-of-exercise rats. Based on the results discussed below, it is evident that intensity and duration of physical activity, the individual endurance capacity, and type of performed exercise are fundamental to establish the magnitude of changes in circulating cytokine concentration. (1) Contrary to chronic stress, 30 or 120 min of aerobic exercise might be insufficient to exert intense perturbations in intracellular milieu so that only small fluctuations in pro- and anti-inflammatory cytokine concentrations occurred. Less profound changes in cytokine profile due to a single session of sprint as compared with 1-week wheel running were also found in mice model [26]. Particularly, when comparing the herein studied cytokine pool, there was a decrease in serum IFNγ in both groups, while in a latter one the authors observed also a decline in IL-6 and G-CSF content [27]. Importantly, the increase in pro-inflammatory cytokines in marathon runners (i.e., ↑ IL-1β, IL-6, IL-8, TNFα) was accompanied by an elevation in cytokine inhibitors (IL-1ra, sTNF-r1, and sTNF-r2) and IL-10, which limits the range of inflammatory response [27]. Indeed, the longer the exercise is and the more muscle mass is involved in the effort, the more considerable changes in serum cytokines should be demonstrated [26]. Therefore, exercise-dependent variations in serum cytokines might be strictly connected with alterations in their production and secretion from skeletal muscles. However, only minimal concordance between these tissues was caused by acute training in mice model, since from 21 altered cytokines’ amount only an increase in IFNγ matched between serum, soleus, and plantaris muscles [26]. (2) According to prior reports, there is a possibility that a crossover effect between exercise bouts after acclimation period attenuated cytokine response. In fact, short-term periods of rest between repeated cycling exercise blunted the changes in circulating cytokine expression in young men [28]. (3) Moreover, a peak in selected cytokine levels (i.e., IL-6, IL-8, IL-10, IL-1β, TNF-α) accrued at the first day of long-distance walking (4 days of training) and afterwards their content decreased [29]. Because of transitory character of stress response, the time frame of highest changes in the level of particular proteins could have occurred either before or after the blood sample collection. For instance, a peak in IL-6 content was observed at the end of exercise session or shortly afterwards (e.g., 30 min or 3 h later) and rapidly reversed to the initial level [30,31], while plasma IL-1β increased 2 h post-exercise [32]. (4) Energetic demands of contracting muscle determines the production of myokines, such as IL-6, that act similarly to stress hormones and mobilize energy substrates [32]. Additionally, IL-6 levels depends on pre-exercise muscle glycogen content, so that its level changes become hard to detect in non-fatiguing exercise [33]. The increased amount of non-esterified fatty acids (NEFA) after an exercise session, most likely reflecting the reduction in triacylglycerol stores in adipose tissue [22], might match the energetic demands of rats in our study, thereby mitigating myokines’ release. Simultaneously, an increase in IL-13 level in F30 group might indicate an activation of Stat3 axis and the development of metabolic adaptations to exercise through transcriptional regulation of mitochondrial biogenesis resulting in endurance capacity improvement [34]. Moreover, plasma level of NEFA and saturated fatty acids (SAT) component are known to modulate immune response [35]. In the control group, several pro- (i.e., IL-1β, IL-7) and anti-inflammatory (i.e., IL-4, IL-10) cytokines correlated, respectively, positively or negatively with the level of SAT, although a majority of the correlations was no longer observed after physical activity. (5) We may also suspect that the level of anti-oxidative defense impacted cytokine concentration based on previous data wherein a single bout of exercise upregulated the Nrf2/HO-1 signal transduction pathway, thereby stimulating antioxidant enzymes and reducing oxidative stress and inflammation [36]. Especially, with increased duration of exercise, we observed a negative correlation between CAT activity and IL-1β, MCP1, MIP-1α, GRO KC, and GM-CSF. (6) Additionally, the kinetic of the immune system and ROS generation responses might differ between each other as exercise-dependent changes in systemic oxidant-antioxidant status occurred approximately 30 min prior to the cytokine generation after cycling exercise in sedentary humans [37]. Mediators of oxidative stress are prerequisites for cytokine release through NADPH oxidase (NOX) and NF-κB activation, which reciprocally stimulate oxidative response [38].

Chemokines control infiltration of monocyte-lineage cells and lymphocytes to tissues, while exercise were shown to be capable of limiting macrophage mobilization in high-fat diet fed mice [39]. Herein, a noticeable impact of an acute bout of exercise on circulating chemokine levels (i.e., ↓ MCP1, MIP-1α, RANTES, and slightly GRO KC) was revealed. In the case of MIP-1α, RANTES and GRO KC most pronounced reductions were observed in groups exercising for 30 min without significant differences between the running speed. MCP1 is representative for these group of cytokines and is involved in macrophage infiltration after skeletal muscle tissue damage [40] and diminished insulin-stimulated glucose uptake into myocytes [41]. MCP1 has been previously classified as myokine being released into the circulation under prolonged exercise conditions, for instance, its increased levels were showed in response to intensive exercise in middle-aged men [42] and in well-trained runners [43]. In contrast, either no changes in plasma MCP1 following maximum progressive swimming in mice [31] or herein noticed decrease were caused by a single exercise session. Recently, a decline in MCP1 and RANTES have been attributed to a reduction in visceral fat tissue depots and observed after regular training in patients with metabolic syndrome [44] and obesity [45,46]. The positive correlations between MCP1, GRO KC, GM-CSF, and VEGF with saturated fatty acids further support also a role of NEFA in the regulation of chemotactic processes. MCP1, GRO KC, MIP-1α, and RANTES exert pro-inflammatory functions and are engaged in the pathophysiology of chronic inflammatory-related diseases [47]. We suppose that exercise-mediated early chemokine reduction limits the acute inflammatory response, although with the increased duration of physical activity this effect declined.

ROS formation induced by physical activity might evoke duel effects, i.e., the infliction of oxidative damage to cellular constituents, and the stimulation of adaptive mechanisms favoring long-term protection [6]. Free radicals are released proportionally to VO_2_max as by-products of mitochondrial oxygen consumption due to ‘electron leakage’ from the electron transport chain in the inner mitochondrial membrane [48]. Other origins of exercise-induced ROS include metal-catalyzed reactions, activated neutrophils and monocytes, NADPH oxidase (NOX) and xanthine oxidase reactions, or arachidonic acid metabolism through the action of lipoxygenases (LOX) and cyclooxygenases (COX) [49]. Simultaneously, enzymatic and non-enzymatic cellular mechanisms synergistically counteract the negative consequences caused by ROS exposure [21]. It is difficult to characterize redox homeostasis solely on the basis of a single biomarker [50,51]. Therefore, in our study, we evaluated enzymatic and non-enzymatic antioxidant barrier, redox status, products of protein and lipid oxidation/glycation, and nitrosative stress. We demonstrated that the activity/concentration of enzymatic (CAT) and non-enzymatic (GSH) antioxidants was significantly higher in rats challenged with exercise. Importantly, GPx neutralizes hydrogen peroxide (H_2_O_2_) at its basal concentrations (Km = 1 µM), while CAT is activated at its higher levels (Km = 2.4 × 10^−4^ M) [52]. Therefore, although not measured directly, it is feasible that supranormal rates of H_2_O_2_ generation represent an early-stage marker of oxidative response to a single bout of treadmill running. The major sources of blood H_2_O_2_ include muscle cells and lungs upon increased blood flow and oxygen uptake, while elevated CAT activity could be attributed to its release from muscles and erythrocytes [53]. A compatible profile of alterations (i.e., ↑ H_2_O_2_ and CAT; ↔ SOD) was shown in skeletal muscle tissue after acute endurance training in mice [54]. H_2_O_2_ arises as a product of superoxide anion (O_2_^•^^−^) reduction and may further inhibit SOD enzymatic activity [55] as well as stimulate p^66Shc^ and forkhead box O3a (FOXO3a) that have a positive feedback on H_2_O_2_ synthesis and redox scavenging system activity, respectively [54]. Although H_2_O_2_ is not a free radical, it may be transformed into highly reactive hydroxyl radical (^•^OH) in the presence of metal catalysts (Haber–Weiss reaction), and cause damage to proteins, lipids, and nucleic acids [56]. The increase in GSH in F30 group, the most abundant intracellular antioxidant, indicates also sufficient ROS defense reserves were maintained during exercise performance. Specifically, exercise increased the expression of muscular γ-glutamylcysteine synthase, the rate-limiting enzyme in GSH biosynthesis [57]. Nevertheless, antioxidants’ level alone does not unambiguously inform about systemic redox status, so that we also evaluated parameters of total antioxidant power of plasma (TAC and FRAP) and the sum of all oxidants in a sample (TOS) [58]. Lack of changes in TAC, TOS, OSI, and FRAP excludes redox homeostasis impairments, instead suggesting that ROS generation was balanced by scavenging systems.

A considerable increment of lipid peroxidation products (LOOH, MDA) content noticed in running groups resembles observations in rats subjected to strenuous exercise (i.e., speed of running: 30 m/min, time: ~60 min, 70–75% VO_2_max) [38] and well-trained humans who underwent acute exercise [8]. It is not surprising, as the increase in both saturated and unsaturated fatty acids was noticed in training animals, as we previously reported [23]. Especially, polyunsaturated fatty acids (PUFAs) with (1Z, 4Z) pentadiene moiety (e.g., linoleic, linolenic, and arachidonic acid) are susceptible for ROS-derived damages and yield the corresponding hydroperoxides, the major primary products of lipid peroxidation [59]. These reactions are self-propagating until the accumulation of different aldehydes, including MDA. MDA easily passes to tissues, being afterwards enzymatically metabolized or reacting with amino acid residues (i.e., lysine, histidine, or arginine) to form adducts with proteins or DNA, leading to biomolecular damages [60]. For instance, lipid peroxidation impacted gene expression and caused loss of membrane integrity, finally resulting in cellular dysfunction [61]. Moreover, the generation of malondialdehyde acetaldehyde (MAA) adducts was responsible for immunogenic reactions [62]. Although we did not notice running time- and intensity-dependent relationship with MDA content, other studies indicate such correlation [63], while adaptations connected with long-term, moderate-intensity exercise enable to diminish lipid peroxidation and prevent tissue damage [64]. In our study we also observed the enhanced amount of AOPP (M30, M120, F30 groups) and AGE (F30 group), i.e., the products of non-enzymatic oxidation and glycation of proteins. Importantly, exercise modality could determine the level of oxidative stress since intermittent running attenuated elevation in AOPP as compared with continuous exercise due to better repair capacities [65]. Products of oxidation and glycation of proteins also enhance the synthesis of pro-inflammatory cytokines/chemokines by inducing NOX activity or stimulating the receptor for advanced glycation end products (RAGE). This occurs during increased accumulation of AGE and AOPP in tissues (e.g., skeletal muscles and blood vessels), especially when the repair mechanisms are weakened (decrease of proteasome activity) [66]. What is more, increased glycoxidation of proteins and their tissue aggregation occurs mainly in hyperglycemic conditions [67]. Therefore, it should not come as a surprise that there is no correlation between the concentration of pro-inflammatory cytokines and redox biomarkers [68]. To date, higher increases in serum RAGE after physical activity were demonstrated for individuals with baseline low-performance levels [69] and in patients with type 2 diabetes [70].

Contractile activity exacerbates NO production through the action of three isoenzymes of nitric oxide synthase (NOS) in a processes stimulated by catecholamines or vascular wall shear stress through AMP-activated protein kinase (AMPK), Akt, and protein kinase A cascade [71], or from nitrates (NO_3_^-^) and nitrites (NO_2_^−^) [72]. In spite of this, the decrease in NO bioavailability in M30 and F30 groups can be explained by its efficient removal through the reaction with oxyhemoglobin and nitrate formation as a consequence of muscle hyperperfusion during exercise [6], although increasing time of training seems to undermine this effect. Additionally, 70–90% of NO is stored as S-nitrosothiols [73], as we observed their elevated amount in F30 group. Interestingly, the precise role of NO appears to be controversial. On the one side, NO competes with O_2_ to bind to cytochrome c oxidase and inhibits enzyme activity in a dose-dependent manner, thereby directing oxygen to nonrespiratory substrates and accelerating ROS production [74] as well as contributing to mitochondrial fragmentation [74], and serves as an important factor for IL-6, IL-8, and TNF-α expression [75]. The co-existence of high O_2_^•^^−^ and NO levels provides the basis for peroxynitrite (OONO^−^) formation, the main culprit of chemical modification of a thiol group and an inhibitor of complexes I and II of the electron transport chain [73]. S-nitrosylation affects proteins’ function, being a powerful inducer of cell death [74]. Nevertheless, upregulation of NOS1 and NOS3 expression, and an increase in NO production, coincided with reduced blood pressure [76] and improved exercise-mediated glucose uptake to skeletal muscles after regular training [72]. In our study, lack of changes in peroxynitrite level, most likely due to low NO availability, negates stimulation of S-nitrosylation of proteins by exercise. Physical activity could also offset the level of muscular S-nitrosylated proteins (i.e., calcium release/uptake proteins, myosine heavy chains) following immobilization [77].

Despite the undoubted advantages, our study also has some limitations. We evaluated only the most frequently analyzed cytokines and redox biomarkers, so we cannot fully characterize the role of inflammation and oxidative stress during physical activity. The next step is to assess the inflammatory and oxidative parameters in the skeletal muscles as well as cardiac tissue.

## 4. Materials and Methods

### 4.1. Animal Experiments

The research was conducted on male Wistar rats (age: two months, body weight: 361 ± 5 g) housed in a stable temperature (20–21 °C ± 2 °C), humidity (40–60%), and 24-h rhythm (12-h light/12-h dark cycle). Free access to a commercial pellet diet for rodents and drinking water was preserved throughout the whole experiment. Animals were habituated for five days to exercise conditions (i.e., 15 min daily of running), and afterwards rats were randomly divided into four equal groups (*n* = 10):

(1) Sedentary control.
(2) Rats running on a treadmill set at +10° incline at the speed of 18 m/min for 30 min (M30).
(3) Rats running on a treadmill set at +10° incline at the speed of 18 m/min for 120 min (M120).
(4) Rats running on a treadmill set at +10° incline at the speed of 28 m/min for 30 min (F30).

The speed of 18 m/min corresponded with approximately 65% of the maximal oxygen uptake (VO_2_max) in rats, whereas 28 m/min was related with approximately 82% of VO_2_max [78]. The total exercise amount was calculated as the product of time and speed of treadmill running (exercise amount = intensity * time), reaching the following values, i.e., M30 = 540 m, M120 = 2160 m, F30 = 840 m. 

Following the exercise, animals were anesthetized by intraperitoneal injection with sodium phenobarbital in a dose of 80 mg/kg of body weight. Blood samples were collected from abdominal aorta and placed into glass tubes (to obtain serum) or glass tubes containing sodium heparin (to obtain plasma).

The protocol of the study was approved by the Ethical Committee for Animal Experiments at the Medical University of Bialystok, Poland (permission number: 72M/2017). 

### 4.2. Cytokine/Chemokine Detection in Serum by ELISA Multiplex

ELISA multiplex was performed to detect the presence of 23 cytokines, according to the manufacturer’s protocol. Briefly, a 96-well plate was washed in a shaker for 10 min (20–25 °C) with a wash buffer solution (100 μL) before being dried and receiving 50 μL of couple beads to each well. Then, standards and samples (1:4 serum dilution) were added into appropriate wells (50 µL) and incubated for 1 h under agitation on a plate shaker. In sequence, the plate was washed twice and 25 μL of detection antibodies were added to the wells for 30 min of incubation under agitation, wrapped with a foil, at 20–25 °C. Afterwards, 50 µL/well of streptavidin-phycoerythrin solution was added. After 10 min, the plate was washed twice, and the beads were resuspended in assay buffer. Finally, the plate was analyzed using the Bio-Plex 200 System (Bio Rad Laboratories, Hercules, CA, USA) for the detection of target cytokines/chemokines amounts in pg/mL.

### 4.3. Determination of Redox Biomarkers

All redox analyses were performed accordingly with previously described procedures [79,80] using reagents purchased from Sigma-Aldrich (Saint Louis, MO, USA). Antioxidant enzymes were analyzed in serum, whereas the non-enzymatic antioxidants, redox status, oxidation products, and nitrosative stress were evaluated in the plasma. The absorbance/fluorescence was measured with a 96-well microplate reader BioTek Synergy H1 (Winooski, VT, USA) and Infinite M200 PRO Multimode Microplate Reader (Tecan, Männedorf, Switzerland). Total protein content was established colorimetrically via the commercial bicinchoninic acid assay (Thermo Scientific PIERCE BCA Protein Assay Kit, Rockford, IL, USA). The results were standardized to 1 mg of total protein.

#### 4.3.1. Antioxidant Barrier

The activity of superoxide dismutase (SOD, EC 1.15.1.1) was estimated spectrophotometrically based on the inhibition rate of adrenaline oxidation to adrenochrome at a wavelength of 480 nm [81]. One unit of SOD activity was defined as the quantity of enzyme inhibiting adrenaline oxidation by 50%.

Catalase (CAT, EC 1.11.1.6) activity was determined spectrophotometrically by measuring hydrogen peroxide (H_2_O_2_) decomposition accompanied by a decrease in the absorbance at 240 nm [82]. One unit of CAT activity was defined as the quantity of the enzyme catalyzing degradation of 1 mM of H_2_O_2_ per 1 min.

The activity of glutathione peroxidase (GPx, EC 1.11.1.9) was assessed using the colorimetric method based on the reduction of organic peroxides by GPx in the presence of reduced nicotinamide adenine dinucleotide phosphate (NADPH) at 340 nm [83]. One unit of GPx activity was assumed to catalyze the oxidation of 1 μmol of NADPH per minute.

The concentration of reduced glutathione (GSH) was established, relying on an enzymatic reaction with 5,5-dithiobis-(2-nitrobenzoic acid) (DTNB), NADPH, and glutathione reductase (GR) [84]. The absorbance of the resulting product was measured at 412 nm.

#### 4.3.2. Redox Status

To assess total antioxidant capacity (TAC), changes in ABTS^+^ (2,2′-azino-bis-3-ethylbenzothiazoline-6-sulfonate) absorbance, under the influence of antioxidants in a sample, were measured at 660 nm [85]. TAC level was calculated from the calibration curve for Trolox (6-hydroxy-2,5,7,8-tetramethylchroman-2-carboxylic acid).

Total oxidant status (TOS) was analyzed spectrophotometrically by measuring the oxidation of ferrous ion to ferric ion in the presence of oxidants in a sample [86]. Ferric ions form a colored complex with xylenol orange in an acidic medium and the level of TOS was calculated from the standard curve for hydrogen peroxide.

Oxidative stress index (OSI) was calculated according to the formula: OSI = [TOS]/[TAC] × 100% [87].

Ferric reducing ability of plasma (FRAP) was determined spectrophotometrically at 593 nm wavelength with the use of 2,4,6-tripyridyl-s-triazine (TPTZ) [88]. FRAP levels were calculated from the calibration curve for FeSO_4_.

#### 4.3.3. Oxidative Damage Products

The amount of plasma advanced glycation end products (AGE) was evaluated spectrofluorimetrically by measuring AGE-specific fluorescence at 350 nm excitation wavelength and 440 nm emission wavelength [89]. Before the assay commenced, plasma samples were diluted (1:5, *v*:*v*) in 0.02 M phosphate-buffered saline (PBS), pH 7.4 [90].

The concentration of plasma advanced oxidation protein products (AOPP) was assessed spectrophotometrically at 340 nm by measuring the total iodide ion oxidizing capacity of the plasma [89]. Prior the assay, plasma was diluted (1:5, *v*:*v*) in 0.02 M PBS [90].

The concentration of lipid hydroperoxides (LOOH) was determined bichromatically at 570/700 nm with the FOX-2 test based on the absorbance of Fe-XO through the reaction of iron (III) ions with xylenol orange (XO) [91]. The absorbance of the Fe-XO complex was measured at a 560 nm wavelength. H_2_O_2_ was used as a standard.

Lipid peroxidation was estimated based on malondialdehyde (MDA) concentration using the thiobarbituric acid reactive substance (TBARS) method [92]. The absorbance of samples was measured at 535 nm wavelength, and 1,1,3,3-tetraethoxypropane served as a standard.

#### 4.3.4. Nitrosative Stress

Nitric oxide (NO) levels were assayed colorimetrically based on the reaction of nitrates with sulfanilamide and N-(1-naphthyl)-ethylenediamine dihydrochloride. The resulting color product exhibited a maximum absorption at the wavelength of 490 nm [93].

The concentration of S-nitrosothiols was measured using the colorimetric method based on the sample incubation with Griess reagent, followed by the reaction with Hg^2+^ ions [94]. The absorbance of the resulting complex was measured at 490 nm.

The amount of peroxynitrite was measured colorimetrically at 320 nm wavelength based on peroxynitrite-mediated nitration of phenol, leading to the formation of nitrophenol [95].

### 4.4. Plasma Free Fatty Acid Content and Composition 

Plasma FFA were analyzed by gas–liquid chromatography, as described previously [23]. Briefly, blood plasma (200 µL) samples were extracted with 2 mL of methanol with 0.01% butylated hydroxytoluene (antioxidant) and 4 mL of chloroform. Moreover, 100 μL of internal standard (heptadecanoic acid (C17:0 FFA) (Sigma-Aldrich, Saint Louis, MO, USA) was added. Thereafter, lipids were separated into specific fractions using thin-layer chromatography (TLC) (Kieselgel 60, 0.22 mm, Merck, Darmstadt, Germany) with a heptane: isopropyl ether: acetic acid (60:40:3, *v*:*v*:*v*) resolving solution. Dried silica plates were sprayed using 3’7’-dichlorofluorescin (0.2% solution in absolute methanol) and specific bands were visualized under ultraviolet light. FFA were transmethylated with BF3/methanol and individual fatty acid methyl esters (FAMEs) were identified and quantified according to the retention times of standards by gas liquid chromatography (Hewlett-Packard 5890 Series II gas chromatograph, HP-INNOWax capillary column; Agilent Technologies, Santa Clara, CA, USA). Total amount of FFA was estimated as the sum of the particular fatty acid species and expressed in nanomoles per milliliter.

### 4.5. Data Analysis and Statistics

The obtained results were analyzed using GraphPad Prism 8 (GraphPad Software, La Jolla, CA, USA). The Shapiro–Wilk test was used to determine the normality of distribution. One-way ANOVA and Tukey’s multiple comparison test were used to compare the studied groups. Whenever the assumptions of normality did not hold, Kruskal–Wallis test followed by Dunn’s test were performed. Multiplicity adjusted *p* value was also calculated. For the consistency of data presentation, median (minimum-maximum) was used. The relationship between particular parameters was assessed based on the Pearson correlation coefficient. The value of *p* < 0.05 was considered statistically significant.

## 5. Conclusions

In conclusion, exercise intervention is a hermetic stress stimulus that induces a synchronous metabolic, immune, and redox response in order to allow a sufficient recovery from challenging conditions. Our data point to chemokines’ reduction in response to a single training session, presumably reflecting initial protection from immune cells’ infiltration to tissues. Although physical effort enhances the enzymatic and non-enzymatic antioxidant barrier, there is an increased oxidation of proteins and lipids. However, the unchanged total antioxidant capacity indicates that cells efficiently managed with stress circumstances. The presented results also underscore that the disturbances in immune and oxidative balance parallel the increase in exercise intensity.

## Figures and Tables

**Table 1:** Plasma content and composition of free fatty acids (FFA) in response to different protocols of treadmill running. The values (nmol/mL) are presented as median (25%–75%) based on 10 independent determinations. ANOVA followed by a post hoc Tukey’s test or Kruskal–Wallis and a post hoc Dunn’s tests were applied to determine significant differences. * *p* < 0.05, exercise group vs. control; ^#^ *p* < 0.05, running speed of 18 m/min vs. the speed of 28 m/min; ^$^ *p* < 0.05, short (30 min) vs. prolonged (2 h) time of exercise session.

FFA	Control	M30	M120	F30
SAT	69.41 (55.90–85.09)	97.08 (86.00–119.84) *	242.07 (159.10–279.02) *^,$^	139.10 (128.14–148.59) *^,#^
UNSAT	73.55 (58.64–98.49)	126.82 (108.18–154.22) *	183.41 (160.34–211.27) *^,$^	170.25 (148.93–178.21) *^,#^
Total	140.18 (116.72–183.53)	224.45 (194.18–274.06) *	401.42 (373.91–458.89) *^,$^	306.85 (289.08–324.31) *^,#^

M30, rats running on a treadmill at the speed of 18 m/min for 30 min; M120, rats running on a treadmill at the speed of 18 m/min for 120 min; F30, rats running on a treadmill at the speed of 28 m/min for 30 min; SAT, saturated fatty acids; UNSAT, unsaturated fatty acids.

## Abbreviations

AGE	advanced glycation end products
AMPK	AMP-activated protein kinase
AOPP	advanced oxidation protein products
CAT	catalase
FOXO3a	forkhead box O3a
FRAP	ferric reducing ability of plasma
G-CSF	granulocyte colony-stimulating factor
GM-CSF	granulocyte-macrophage colony stimulating factor
GPx	glutathione peroxidase
GRO KC/CXCL1	growth-regulated oncogene/keratinocyte chemoattractant/chemokine (C-X-C motif) ligand 1
GSH	reduced glutathione
HO-1	heme oxygenase-1
IFNγ	interferon γ
IL	interleukin
IL-1ra	interleukin-1 receptor antagonist
JNK	c-Jun N-terminal kinases
Km	Michaelis–Menten constant
LOOH	lipid hydroperoxides
MAA	malondialdehyde acetaldehyde
MAPK	mitogen activated protein kinase
MCP1/CCL2	monocyte chemoattractant protein 1/chemokine (C-C motif) ligand 2
M-CSF	macrophage colony-stimulating factor
MDA	malondialdehyde
MIP-1*α*/CCL3	macrophage inflammatory protein-1α/chemokine (C-C motif) ligand 3
MIP-3α/CCL20	macrophage inflammatory protein-3α/chemokine (C-C motif) ligand 20
NADPH	nicotinamide adenine dinucleotide phosphate
NEFA	non-esterified fatty acids
NF-κB	nuclear factor κB
NO	nitric oxide
NOS	nitric oxide synthase
Nrf2	nuclear factor-like 2
RAGE	receptor for advanced glycation end products
RANTES/CCL5	regulated on activation, normal T-cell expressed and secreted/chemokine (C-C motif) ligand 5
SOD	superoxide dismutase
TNFα	tumor necrosis factor α
VEGF	vascular endothelial growth factor
